# Cervical vagal nerve schwannoma induced arrhythmia: a rare case report and literature review

**DOI:** 10.1186/s12883-022-03016-2

**Published:** 2022-12-14

**Authors:** Pin Ye, Changhuai He, Yunfei Chen, Hongxiao Wu, Yiqing Li, Chuanqi Cai, Ping Lü

**Affiliations:** Department of Vascular Surgery, Union Hospital, Tongji Medical College, Huazhong University of Science and Technology, Wuhan, 430022 China

**Keywords:** Cervical vagal nerve schwannoma, Nerve schwannoma, Vagus nerve stimulation, Arrhythmia, Surgical intervention

## Abstract

**Background:**

Schwannomas are benign tumors deriving from the sheath of cranial and peripheral nerves. The vagus nerve is comprised of a complex neuro-endocrine-immune network that maintains homeostasis, most tracts of it play a role in parasympathetic activity. We present an example of a rare cervical vagal schwannoma case accompanied by arrhythmia.

**Case presentation:**

A 35-year-old female patient with a left cervical vagus schwannoma and ventricular arrhythmia underwent schwannoma resection in the operating room. The patient’s suppressed heart rate increased after tumor removal, and the cardiac rhythm returned to normal postoperatively. Pathological examination demonstrated the diagnosis of schwannoma.

**Conclusions:**

This case explains the link between the vagus nerve and the cardiovascular system, proving that a damaged cervical vagus nerve can inhibit the heart rate and lead to arrhythmias, and eventually requiring surgical intervention.

## Introduction

Schwannoma is the most common type of peripheral nerve tumor usually arising from Schwann cells [1]. Vagal schwannoma (VS) could present as a solitary, asymptomatic, and slow-growing benign tumor in the neck [2]. The vagal nerve (VN), the longest cranial nerve, performs motor and sensory functions in afferent and efferent aspects and controls the cardiovascular, respiratory, and digestive systems [3]. Symptoms may occur if the mass invades and compresses the surrounding blood vessels, nerves and tissues. It can be challenging for the surgeon to diagnose. Pre-operative imaging provides important information about the location, size and nature of the mass. The origin of the mass can be visualised intraoperatively and the diagnosis confirmed by pathology. Surgery is currently the most effective treatment, but the discussion of intraoperative and postoperative complications due to nerve damage has been a hot topic of research, which requires the collaboration of imaging doctors, anesthetists and surgeons to develop a comprehensive treatment plan for the patient. Herein we present a rare cervical vagal schwannoma (CVS) case accompanied by arrhythmia. The patient experienced rapid recovery in her heart rate and rhythm postoperatively. Identification of VS and early surgical intervention could be crucial in preventing this arrhythmia complication. It is hoped that this case and the related literature review will give imaging doctors, anesthetists and surgeons new insight into this type of disease and provide a little experience for subsequent treatment.

### Case presentation

**Fig. 1:**
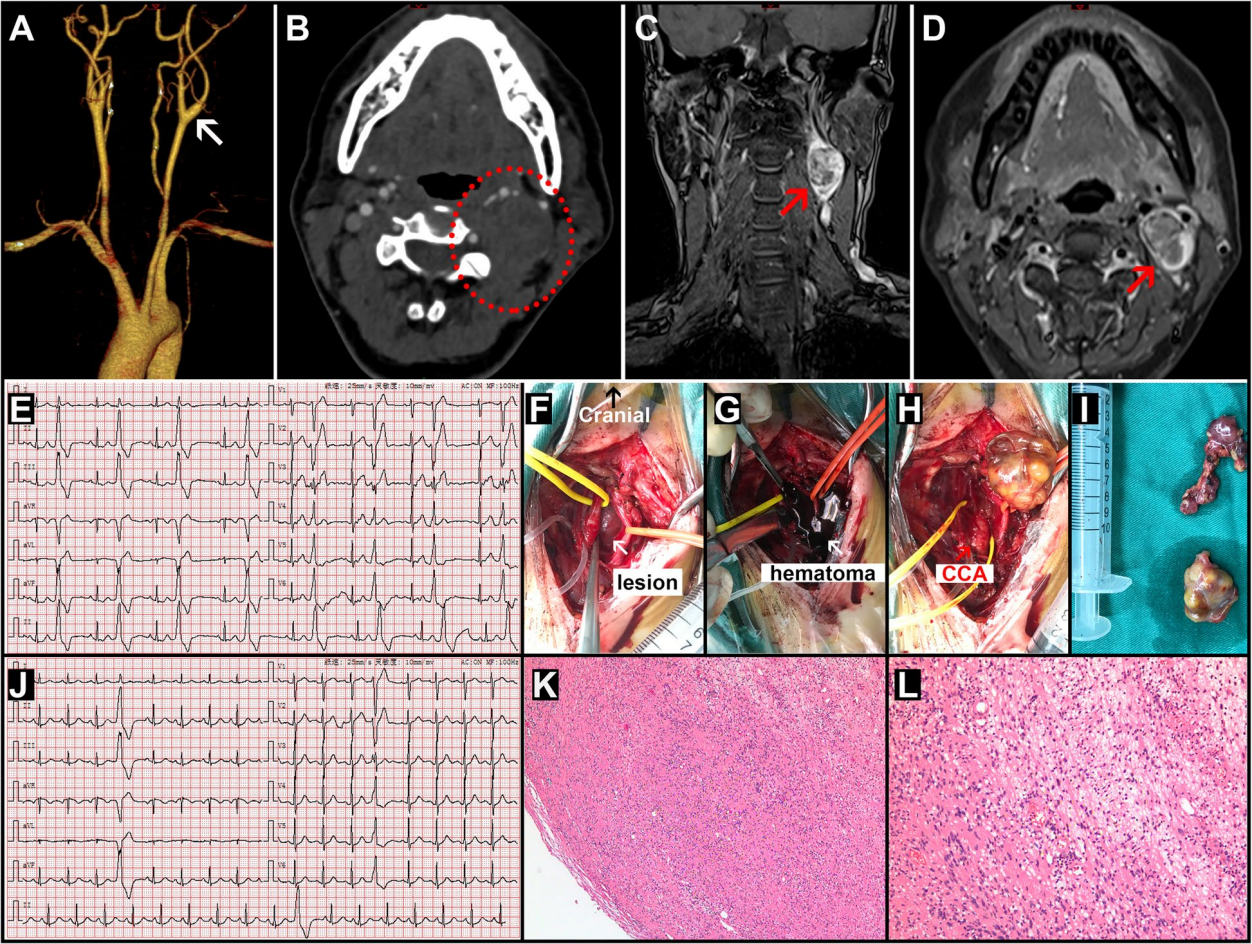
Case data. (**A–B**) Preoperative computed tomography angiography (CTA) presented the lesion located in the bifurcation of the left common carotid artery without abundant blood supply in the white arrow and dashed circle area. (**C-D**) Preoperative magnetic resonance imaging (MRI) presented the non-homogeneous lesion in the red arrow area. (**E**) Preoperative electrocardiogram (ECG) presented a sinus rhythm with frequent premature ventricular beats as a duality. (**F-I**) The mass and hematoma were totally removed, and the vagal nerve was intact. (**J**) Postoperative ECG presented a sinus rhythm with occasional premature ventricular beats. (**K-L**) HE section presented the spindle cells arranged in small bundles.

A 35-year-old woman was referred to our department for a palpable mass in the left cervical area. On admission, she suffered from heart palpitations for the past year. Moreover, she was diagnosed with idiopathic arrhythmia from her electrocardiogram (ECG) examination without any positive findings from her cardiac ultrasound scan. She had no other histories of diabetes mellitus, hypertension, or cerebral infarction. On examination, there was a soft, smooth-surfaced mass in the left middle cervical region, measuring 3 cm × 2 cm, without color changes in the skin. Her cardiac discomforts worsened as a result of palpating the mass. Further investigation, including common carotid artery computed tomography angiography (CTA) and magnetic resonance imaging (MRI), revealed a solid mass located in the left common carotid artery (LCCA) bifurcation without abundant vascularity **(**Fig. 1A-D). Based on the results of CTA and MRI, we had an in-depth discussion with the radiologist and concluded that the mass was a shwannoma. Since the mass affected the heart, we communicated with the patient and decided to surgically remove it. The patient was informed of the various risks associated with surgery, mainly complications from nerve damage. Her preoperative ECG presented a sinus rhythm with frequent premature ventricular beats as a duality, and her heart rate was around 70 bpm (Fig. 1E). Under general anesthesia, mass resection surgery was performed. Intraoperative arrhythmia was evident and was resistant to lidocaine administration. There were markedly tumid lymph nodes surrounding LCCA with dilated left internal jugular vein. The mass was exposed posterior to LCCA bifurcation and adjacent to the vagal nerve (Fig. 1F). Due to the strange dark color of this mass, we punctured it with a syringe needle and discovered that it contained hematoma. We then cut this mass with a longitudinal incision to drain around a 3 mL hematoma (Fig. 1G). In perfect coordination with the anaesthetist, the vagal schwannoma (VS) mass was totally peeled off from the underlying nerve fibers, leaving the vagal nerve was intact (Fig. 1H). Unexpectedly, her arrhythmia was noticeably alleviated post-decompression. Postoperatively, the fresh mass was heterogeneous (Fig. 1I).

Her heart rate increased to approximately 95 bpm immediately following surgery. Her postoperative ECG revealed a sinus rhythm with occasional premature ventricular beats (Fig. 1J), and her palpitation symptom was clearly relieved. The pathological examination confirmed the diagnosis of schwannoma and the spindle cells were arranged in small bundles (Fig. 1K-L**)**. Furthermore, abundant S100 (+), SOX 10 (+) cells, and partial Ki67 positive cells (positive index: 5%) were observed in immunohistochemical staining (data not shown). She was discharged 5 days after surgery without experiencing any symptoms or complications. There was no sign of recurrence at the 1 year follow-up.

## Discussion and conclusions

At present, there are no epidemiological features associated with Cervical vagal nerve schwannoma based on clinical reports, nor are there genetic and molecular mechanistic studies associated with it. A recent case report on CVS demonstrated that it was initially misdiagnosed as a malignant lymph node on ultrasound. However, intraoperative pathology revealed a vagal schwannoma, highlighting the challenges in diagnosis [4].Schwannoma may be completely asymptomatic due to its particular position and proximity to important anatomical structures. It is often seen clinically as a neck lump and is therefore difficult to differentiate on physical examination and is differentially diagnosed from Paraganglioma, Branchial cleft cyst, Inflammatory, adenopathies, Malignant lymphoma, Metastatic cervical lymphoadenopathies, Submandibular salivary gland tumours and Carotid artery aneurysm. Schwannoma in the neck appears as a well-defined envelope that grows at a rate of 2.5–3 mm per year, destroying the vagal nerve (VN) and surrounding structures, which may induce additional symptoms [5], including hoarseness, dysphagia, airway obstruction, dyspnea, and pain, as reported in other published cases [6]. Diagnostic imaging techniques are critical. Ultrasound, computed tomography, and magnetic resonance imaging are the most popular examination methods for this tumor, not only for lesion position determination but also for blood supply evaluations. The cell of origin for schwannomas is one with a basement lamella. We believe that Schwann cells and perineural fibroblasts are the most likely precursors of schwannomas, respectively. Microscopically, tumor type displays well-differentiated, fusiform, spindle cells, without frequent mitoses. Therefore, pathological examination is the gold standard for diagnosis. Recently, a trend towards malignant transformation of cervical vagal schwannomas has been reported, and surgical treatment remains an effective option in order to relieve the compression symptoms of the mass [7]. However, there are some risks associated with surgical treatment, which can affect the state of the vagus nerve. Our case represents an important contribution to the literature regarding cervical vagal schwannoma (CVS). Currently there are two main types of surgery, enucleation and resection, with enucleation being more effective in preserving nerve function and resection being more effective in avoiding recurrence [5, 8]. We systematically reviewed all CVS cases from 2015 to the present, including 39 patients from 15 studies in this literature review. (Table 1 for patient clinical information) We found that the male/female ratio was 0.7 in 39 patients, the age at diagnosis of the disease was 48 ± 12 (*n* = 39). Of these patients, 31% (*n* = 12) were asymptomatic and presented solely with a neck lump, while 69% (*n* = 27) presented with symptoms due to an enlarged lump that invaded surrounding blood vessels, nerves and tissues. Surgical treatment was chosen in all the reported cases and the follow-up results in 37 patients showed that 84% (*N* = 31) of the patients recovered well after surgery,8% (*N* = 3) had complications related to nerve damage and 8% (*n* = 3) presented with local recurrence after enucleation (11.6 ± 4 months). Therefore, surgical resection seems to be a better option, but care should be taken to avoid nerve and vascular damage and complications. To the best of our knowledge, this case report represents the first example of CVS complicated by arrhythmia due to damage to the vagus nerve (VN). VN, the 10th cranial nerve, affects numerous organ systems and regions of the body, such as the heart, tongue, pharynx, and gastrointestinal system [3]. The reduced vagal tone has been associated with a higher risk of death. In addition, we have found in published case reports that intraoperative manipulation of the vagus nerve tends to induce cardiac and circulatory fluctuations in patients, resulting in bradycardia and reduced blood pressure [10, 21].The anaesthetist should assess the vocal cords preoperatively as well as inform the patient of possible postoperative neurological complications and avoid the use of anaesthetic drugs that cause bradycardia [10]. It has been reported that the left vagal nerve has a greater influence on atrioventricular conduction [22], which is consistent with our case. VN is vital, and when schwannomas compress it, the parasympathetic tone could be decreased, and sympathetic activity could be increased, resulting in loss of the synergistic protective effect on the heart. A case of intraoperative cardiac arrest has been reported by DK Mukherjee [23]. In the present case, we demonstrated the effects of vagal nerve stimulation on the cardiovascular system.

**Table 1:** Summary of the findings in reported Cervical vagal nerve schwannoma cases

Author	NO.	Age(years or mean ± SD years)	Schwannoma diameter (mm or mean SD ± mm)	Gender	Site(Right or Left)	Symptoms	Treatment	Follow up	Note
Carroll [9]	1/1	31	62 × 55	Male	L	Swelling	Surgery	Good	
Pankratjevaite [4]	1/1	55	30 × 20	Female	L	Swelling	Surgery	Good	
Mahajan [7]	1/1	41	10 × 90	Male	L	Swelling, difficulty swallowing and hoarseness	Surgery	Good	
Saini [10]	1/1	34	50 × 40	Female	L	Swelling, hoarseness and dizziness	Surgery	Hoarseness	Bradycardia and hypotension during surgery
Keshelava [11]	1/1	50	–	Male	L	Swelling	Surgery	Good	Compression of internal carotid artery
Harhar [12]	1/1	76	30 × 30	Male	R	Swelling and dizziness	Surgery	Good	
Xu [13]	1/1	31	68	Female	L	Swelling and pain	Surgery	Hoarseness	
Walters [14]	1/1	15	35 × 60	Female	R	Swelling	Surgery	Good	Postoperative apnea
Kumar [15]	1/1	18	–	Female	R	Swelling	Surgery	Horner syndrome	Sympathetic nerve injury
Cukic [16]	1/1	60	86 × 70	Female	L	Swelling	Surgery	Good	
Ansari [17]	1/1	23	30 × 20	Male	R	Swelling	Surgery	Good	
Kamath [18]	2/2	48 ± 9	30 ± 10	Male/Female(1, n = 2)	L/R(1, n = 2)	Swelling and difficulty swallowing	Surgery	–	
Shinohara [19]	1/1	52	30 × 40	Male	L	Swelling	Surgery	Good	
Sandler [20]	3/3	33 ± 9	30 ± 4	Male/Female(0.5, n = 3)	R	Swelling, Cough, Pain	Surgery	Good	
Giulio [8]	22/22	49 ± 13	37 ± 14	Male/Female(0.6, *n* = 22)	–	20 patients, Vocal cord palsy; 2 patients, No symptoms	Surgery	Significant voice improvement	3 patients presented after enucleation (3/22): local recurrence

NO., number of patients; L, left; R, right; −, unknow; Schwannoma can clinically affect the patient’s circulatory stability through invasion of the vagus nerve. Surgical resection is a durable and effective way of treating CVS patients, but close protection of the patient’s nerves and blood vessels is required to avoid complications

## Data Availability

The datasets used and analysed during the current study are available from the corresponding author on reasonable request.

## Abbreviations

VS: vagal schwannoma
VN: vagal nerve
CVS: cervical vagal schwannoma
ECG: electrocardiogram
CTA: computed tomography angiography
MRI: magnetic resonance imaging
LCCA: left common carotid artery
